# Resting and Physical Activity Energy Expenditure Across an Altitudinal Gradient: An Adjusted Analysis

**DOI:** 10.3390/oxygen6030019

**Published:** 2026-07-14

**Authors:** Margot Evelin Bernedo-Itusaca, Shantal Cutipa-Tinta, Judith Marie Merma-Valero, Tatiana Milagros Cruz-Riquelme, Sintia Tatiana Flores-Coila, Mahely Adriana Coa-Coila, Claudia Alejandra Coriman-Cuentas, Mayra Anay Condori-Apaza, Ruth Karina Pérez-Flores, Rocío del Rosario Ramos Allazo, Max Smith Abollaneda Amao, Alberto Alcibiades Salazar Granara, Kevin Pacheco-Barrios, Moua Yang, Ginés Viscor, Ivan Hancco Zirena

**Affiliations:** 1 Facultad de Medicina Humana, Universidad Nacional del Altiplano, Puno 21000, Peru; 2 ACEM (Asociación Científica de Estudiantes de Medicina), Universidad Nacional del Altiplano, Puno 21000, Peru; 3 Facultad de Medicina Humana, Universidad Nacional de San Agustín de Arequipa, Arequipa 04000, Peru; 4 Centro de Investigación en Medicina de Altura (CIMA), Facultad de Medicina Humana, Universidad de San Martín de Porres, Lima 15001, Peru; 5 Neuromodulation Center and Center for Clinical Research Learning, Spaulding Rehabilitation Hospital and Massachusetts General Hospital, Harvard Medical School, Boston, MA 02115, USA; 6 Unidad de Investigación para la Generación y Síntesis de Evidencias en Salud, Vicerrectorado de Investigación, Universidad San Ignacio de Loyola, Lima 15023, Peru; 7 Bloodworks Northwest Research Institute, Seattle, WA 98102, USA; 8 Division of Hematology and Oncology, Department of Medicine, University of Washington School of Medicine, Seattle, WA 98195, USA; 9 Sección de Fisiologia, Departament de Biologia Cel·lular, Fisiologia i Immunologia, Facultat de Biologia, Universitat de Barcelona, E-08028 Barcelona, Spain

**Keywords:** excessive erythrocytosis, hypoxia, high altitude, resting energy expenditure, physical activity

## Abstract

**Introduction:**

Survival at high altitudes requires efficient energy management. Although hypobaric hypoxia alters thermodynamic efficiency, the independent impact of altitude versus demographic factors on basal and exertional caloric costs remains uncertain. We evaluated these variables in chronic residents across four Peruvian altitudes.

**Methodology:**

A cross-sectional study was conducted involving 141 healthy adults (aged 18–38 years) residing in Lima (154 m), Arequipa (2335 m), Puno (3827 m), and La Rinconada (5100 m). Resting energy expenditure (REE) and physical activity energy expenditure (PAEE) were estimated using continuous photoplethysmography; PAEE was assessed following the 6-min walk test (6MWT). Hemodynamic parameters, oxygen saturation (SpO_2_), and hemoglobin (Hb) levels were evaluated. An analysis of covariance (ANCOVA) was employed to adjust metabolic variations for age, sex, and body mass index (BMI).

**Results:**

Unadjusted data demonstrated a progressive increase in REE and PAEE proportional to altitude. However, the ANCOVA revealed that the independent effect of the city of residence was no longer statistically significant after adjusting for anthropometric and demographic covariates. Physiologically, the SpO_2_ deficit imposed a high metabolic demand (an increase of approximately 1.29 kcal in REE for every 1% drop in SpO_2_). Hb concentrations above 18 g/dL were associated with an exponential increase in caloric cost driven by blood hyperviscosity. A positive correlation was identified between Hb levels and energy expenditure (EE), which proved to be statistically stronger in the female cohort. Under extreme hypoxia conditions (5100 m), men exhibited a significantly higher PAEE (50.60 ± 10.17 kcal vs. 40.78 ± 5.21 kcal). Despite the increased biological effort, mechanical performance in the 6MWT remained constant across cities.

**Conclusions:**

There is no independent relationship between REE and the altitude of residence. The initial unadjusted relationship between altitude and EE was negated by covariates, particularly body mass index (BMI). The preservation of functional capacity at the expense of the energy economy underscores the profound physiological burden of Andean acclimatization.

## 1. Introduction

Human survival and performance in high-altitude environments depend on the efficient management of energy resources [1]. Total energy expenditure (TEE), comprising basal metabolism, thermogenesis, and energy expenditure (EE) from physical activity, represents the “currency” with which the organism negotiates its adaptation to the environment [2,3]. In young and active populations, the metabolic system possesses remarkable plasticity; however, when subjected to extreme variations in barometric pressure, homeostatic mechanisms must prioritize the maintenance of vital functions, which often results in altered thermodynamic efficiency and an increase in the caloric cost of daily tasks [4,5].

Exposure to altitude introduces hypobaric hypoxia as a disruptive factor in metabolic efficiency [6,7]. As a consequence of the decreased partial pressure of oxygen (PO_2_) and oxygen saturation (SpO_2_), the human organism activates a cascade of physiological responses mediated by the sympathetic nervous system through a persistent adrenergic stimulation [8,9]. This response, while necessary to preserve organs function, significantly increases the basal metabolic cost due to the increased cardiorespiratory workload [10]. In this scenario, hypoxia could act as an “accelerator” of EE, which has positioned altitude as a critical natural model for studying metabolic adaptability and its influence on the control of chronic pathologies linked to energy balance, such as obesity and insulin resistance [11,12].

A determining factor in the magnitude of these adaptations is the hematological profile and the time span of residence [13]. While some high-altitude populations have developed genetic adaptations to optimize oxygen transport [7], the Andean phenotype typically exhibits a response characterized by excessive erythrocytosis [14]. This increase in red blood cell mass (RBC) and hemoglobin (Hb) raises blood viscosity and alters nutrient kinetics delivery, generating a differential impact on the caloric expenditure of permanent residents [10]. Analyzing this phenomenon across different levels (from low (0–2000 m) to high altitudes (>3000 m)) would allow us to observe how SpO_2_ and hematological compensation modulate the efficiency with which the body uses its energy reserves both at rest and during low intensity exertion [15].

Despite advances in altitude physiology, evidence regarding metabolic requirements at the absolute frontier of human tolerance remains sparse, particularly among young, active populations. La Rinconada, Peru (5100 m), represents a unique worldwide location to evaluate these costs under conditions of chronic, severe hypoxia [7,16]. This study aims to characterize and compare variations in resting energy expenditure (REE) and physical activity energy expenditure (PAEE), assessed via the 6-min walk test [17], across a Peruvian altitudinal gradient: Lima (154 m), Arequipa (2335 m), Puno (3827 m), and La Rinconada (5100 m). We hypothesized that increasing altitude is associated with a significant escalation in EE, primarily driven by the physiological demands elicited by hypobaric hypoxia exposure.

## 2. Materials and Methods

### Ethical aspects:

Participants voluntarily agreed to take part in the study after each procedure and the potential usefulness of the results had been thoroughly explained, and they subsequently signed an informed consent authorizing the corresponding measurements.

This study followed the guidelines of the Declaration of Helsinki on research involving human subjects and was approved by the USMP Ethics Committee with the International Registry Federalwide Assurance (FWA) for the Protection of Human Subjects for International No. 00015320. U.S. Department of Health and Human Services (HHS) Registration of an Institutional Review Board (IRB) IRB No. 00003251.

### Study Population:

The study population consisted of 141 healthy subjects: 70 women and 71 men. 40 subjects (20 women and 20 men) were residing in Lima, 40 (20 women and 20 men) in Arequipa, 42 (21 women and 21 men) in Puno, and 19 (9 women and 10 men) in La Rinconada. The age range of participants was between 18 and 38 years old. They were selected according to predefined inclusion criteria: individuals in apparent good health who had resided in their respective localities for at least one year, thereby ensuring chronic exposure to local environmental and hypoxic conditions. To estimate the level of habitual physical activity (sedentary, light, moderate, or intense), the Spanish short version of the International Physical Activity Questionnaire (IPAQ-SF) was administered, selecting for the study those classified as having moderate physical activity [18–20]. Thus, all participants were able to complete the six-minute walk test (6MWT), undergo basal metabolic and vital sign measurements, and supply the required demographic information. Exclusion criteria included refusal to participate; any limitation preventing engagement in light-to-moderate physical activity; the onset of discomfort leading to withdrawal during assessments; or inability to complete the full set of required measurements.

### Procedure:

The assessments were conducted in a controlled environment between 8:00 and 10:00 a.m. [21]. Similar environmental conditions were maintained in all the study cities, and participants wore light clothing. Specifically, the ambient temperature was kept between 10 and 20 °C, and the terrain was completely flat.

Before the assessment, subjects were required to: refrain from vigorous exercise for 24 h and moderate exercise for at least 2 h prior to the evaluation; observe a 5- to 6-h fast to minimize the thermic effect of food; and completely abstain from stimulants (caffeine, nicotine) and alcohol for 12 to 24 h [22,23]. Finally, a 10-min period of absolute rest was mandated in a quiet environment before measurement to ensure stability in oxygen consumption (VO_2_) and reduce emotional stress [24].

Body weight was recorded using a Tanita BC 545N segmental body analysis scale (Tanita, Tokyo, Japan), which employs the bioelectrical impedance method [25]. Height was measured with a DETECTO 2391 stadiometer (Cardinal/Detecto Scale, Webb City, MO, USA) [26].

To determine Hb and hematocrit (Hct) levels, blood samples were obtained from the middle fingertip. A capillary puncture was performed using a sterile lancet, the first two drops were discarded, and the third drop was then collected to fill the microcuvette. Hb levels were assessed using a portable Hb 201+ hemoglobinometer (HemoCue AB, Ängelholm, Sweden) employing the azidimethemoglobin method within a measurement range of 0 to 25.60 g/dL. Hct measurements were performed using a Hemata Stat II microcentrifuge (EKF Diagnostics, Boerne, TX, USA).

The Kalenji HR500 smartwatch (Decathlon, Villeneuve-d’Ascq, France), worn on the right wrist in contact with the skin, was employed to measure chronotropic response (CR) and EE. To determine REE, a continuous 5-min period of physiological stability, or steady state, was first identified after acclimatization. This steady state was confirmed if the heart rate’s (HR) coefficient of variation (*CV*) was ≤10%, calculated using the formula presented in Equation (1).

(1) $$\text{CV}=\left(\frac{\sigma }{\bar{X}}\right)\times 100$$

where *σ* represents the standard deviation and $\bar{X}$ the mean HR within that interval. The mean heart rate obtained during this steady-state period was subsequently used as the reference value for analysis.

REE was assessed over a 20-min period after participants reached a physiological steady-state while resting, with no physical exertion. Caloric expenditure was calculated in Kcal/min. For the measure of physical exertion, the 6MWT was administered following the American Thoracic Society (ATS) protocol. Participants were instructed to cover the greatest possible distance on a 60-m, obstacle-free course [27]. Continuous HR monitoring at the wrist was performed using the watch’s PPG sensor, allowing for REE immediate estimation upon completion of this test.

For kilocalorie consumption calculation, the device’s algorithm combines the chronotropic response with the subject’s anthropometric variables (age, sex, weight, and height) to estimate basal metabolic rate and EE from physical activity [28].


$$\text{EE}=\text{gender}\times \left(-55.0969+0.6309\times \text{heart}\;\text{rate}+0.1988\times \text{weight}+0.2017\times \text{age}\right)+\left(1-\text{gender}\right)\times \left(-20.4022+0.4472\times \text{heart}\;\text{rate}-0.1263\times \text{weight}+0.074\times \text{age}\right)$$

Vital signs were measured before and after REE and PAEE measurements. Systolic and diastolic blood pressure (SBP and DBP) and HR were measured using a Riester Ri-Champion adult digital upper arm sphygmomanometer (Rudolf Riester GmbH, Jungingen, Germany), with a measurement range of 30 to 280 mm Hg and an HR range of 40 to 200 beats per minute. SpO_2_ was measured using a Nellcor^®^ Oximax^®^ N-65 portable pulse oximeter (DigiCare Biomedical, Boynton Beach, FL, USA) with a saturation resolution of 1% and an HR range of 30 to 235 beats per minute.

### Statistical Analysis:

Data normality was verified using the Kolmogorov–Smirnov test. Descriptive statistics were expressed using measures of central tendency and dispersion. Bivariate comparisons across altitude groups were performed using Student’s *t*-test, one-way ANOVA, and Kruskal–Wallis tests, with Tukey’s Honestly Significant Difference (HSD) applied for post-hoc analyses. Associations between physiological variables were assessed using Pearson correlation coefficients and simple linear regression models. To account for potential confounders and isolate the independent effect of altitude on EE, an analysis of covariance (ANCOVA) was employed, adjusting for age, sex, and body mass index (BMI) as covariates. All statistical analyses were performed using Python version 3.0 and the threshold for statistical significance was set at *p* < 0.05.

## 3. Results

The subjects studied showed similar anthropometric characteristics (Table 1), with a slight increase in age as altitude increased. Regarding Body Mass Index (BMI), mean values indicating overweight (≥25 kg/m^2^) were observed in men from Arequipa and in both sexes at 5100 m, while average weight was normal in the other groups (Table 1).

Regarding oxygenation and transport parameters, a progressive and statistically significant decrease in SpO_2_ was observed with increasing altitude in both sexes (*p* < 0.001 for both). Conversely, Hb and Hct concentrations showed a significant escalation (*p* < 0.001) directly proportional to the altitude of residence, reaching peak values in La Rinconada for both women (17.5 ± 1.6 g/dL) and men (21.0 ± 2.2 g/dL). (Table 2) Hemodynamic responses exhibited distinct sex-based patterns across the altitudinal gradient. In women, HR increased significantly (*p* < 0.001), while mean arterial pressure (MAP) showed significant altitude-dependent variations (*p* = 0.004), characterized by an initial decline at moderate altitudes followed by a return to near-baseline levels at 5100 m (84.5 ± 8.3 mmHg). This significant variation in women was also observed in SBP (*p* = 0.042) and DBP (*p* = 0.002). In contrast, while men exhibited a significant increase in HR (*p* = 0.007), no statistically significant changes were observed in their MAP (*p* = 0.486), SBP (*p* = 0.610), or DBP (*p* = 0.521) as a function of altitude (Table 2).

Physical activity levels, assessed using the IPAQ questionnaire, classified the entire sample into the light or moderate intensity categories, registering values below 1400 METs (Table 3). When the results were stratified by sex, it was observed that men presented slightly higher levels of physical activity than women. However, in both groups, the values remained constant regardless of the city of residence, showing no statistically significant differences associated with altitude (*p* = 0.997) (Table 3).

Resting HR showed a predictive value for REE of 0.05 kcal/min per bpm. When stratified by sex, the coefficients remained identical for women (R = 0.71, β = 0.05) and men (R = 0.65, β = 0.05), indicating a uniform contribution of HR to the EE estimate regardless of gender.

Regarding PAEE, the increase per bpm during physical activity was 0.08 kcal/min. Sex-stratified analysis showed consistent results, yielding a coefficient of β = 0.08 in women and β = 0.09 in men, reflecting the direct proportionality between HR and PAEE in the estimation model.

In the unadjusted analysis, REE appeared to show a progressive increase across the altitudinal gradient, reaching peak values in La Rinconada for women (2.08 ± 0.82 kcal/min) and men (2.48 ± 0.97 kcal/min). However, this unadjusted finding is misleading, as the observed increase is, at least in part, attributable to the higher body mass index (BMI) recorded at greater altitudes, which accounts for the variations in resting metabolic demand. While sex-based patterns were generally consistent at most altitudes (*p* > 0.05), a significant divergence was noted in Arequipa, where women demonstrated significant higher metabolic demands than men (*p* = 0.029) (Table 4).

To account for demographic and anthropometric confounders and isolate the independent effect of altitude on REE, an ANCOVA model was applied. After adjusting the analysis for age, sex, and body mass index (BMI) as covariates, including their interactions, the independent effect of the city was not statistically significant (F = 0.400, *p* = 0.753). Similarly, no significant main effects were observed for age (*p* = 0.488), sex (*p* = 0.808), or BMI (*p* = 0.656). These findings suggest that the discrepancies observed in gross EE may be primarily linked to the anthropometric and demographic particularities of the evaluated subjects, rather than altitude in isolation (Table 5).

Similarly, to account for potential confounders during physical exertion, an ANCOVA was performed for PAEE adjusting for age, sex, and BMI. While the overall model explained 49.1% of the variance (R^2^ = 0.49, F = 8.05, *p* < 0.001), the main effect of city of residence on PAEE was not statistically significant after adjustment (F = 2.40, *p* = 0.070). Similarly, age (*p* = 0.222), sex (*p* = 0.726), and BMI (*p* = 0.756) showed no independent significant effects (Table 6).

Notably, despite the increased metabolic cost and cardiac effort, reflected in a significantly higher post-activity HR at altitude (107.63 bpm in La Rinconada vs. 92.08 bpm in Lima), no significant differences were found in the distance covered in the 6MWT between these cities (*p* = 0.830) (Table 7).

Conversely, under conditions of physical activity in extreme hypoxia (La Rinconada), a significant difference was observed (*p* = 0.018), with males showing a substantially higher PAEE (8.43 ± 1.69 kcal/min) compared to females (6.80 ± 0.87 kcal/min) for the same physical stimulus. In the other cities, PAEE showed no differences related to sex (Table 7).

**Figure 1.**
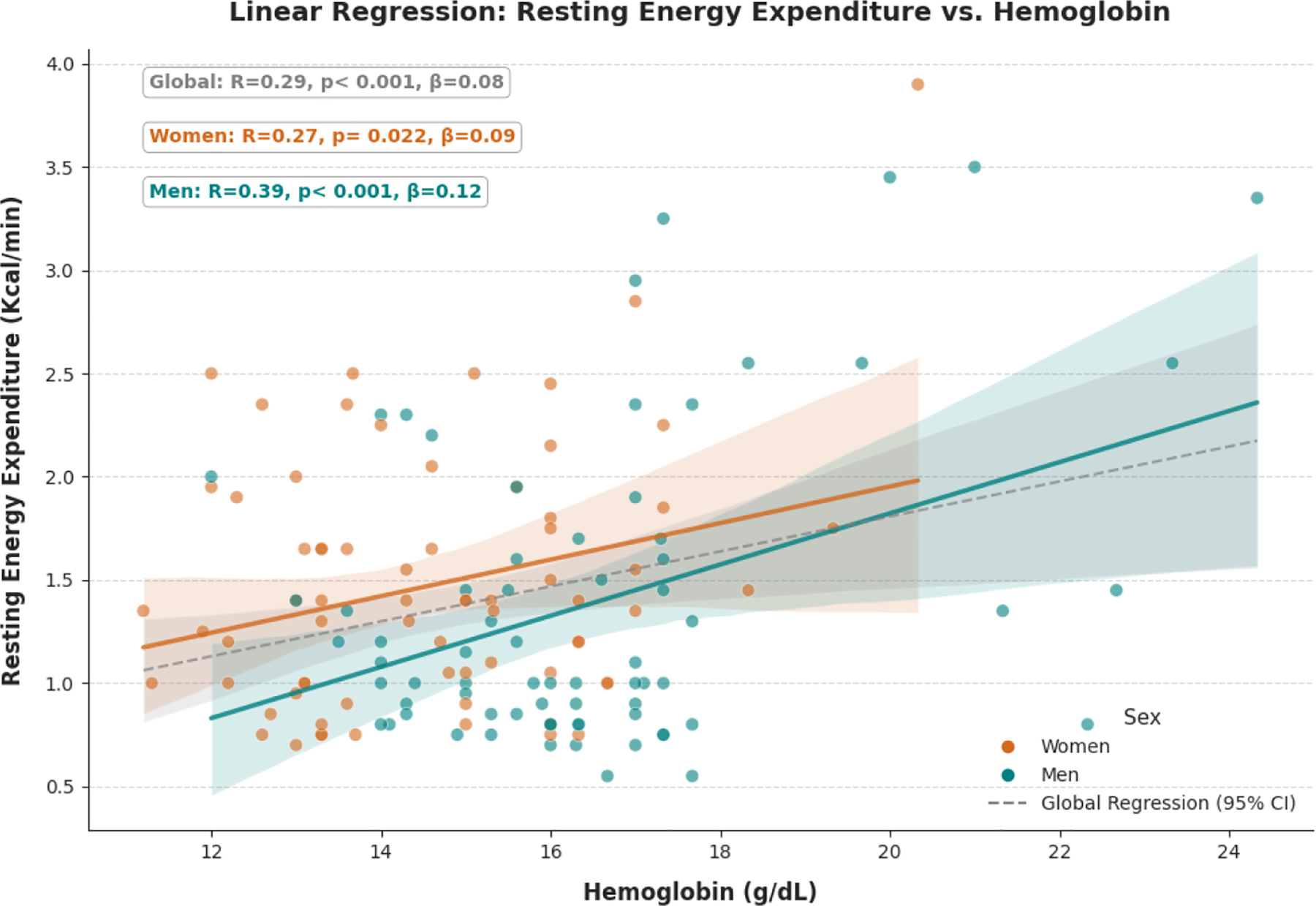
Linear Regression of Resting Energy Expenditure vs. Hemoglobin Levels, stratified by sex. Data are coded by color: women (orange) and men (turquoise), along with an overall regression line (gray dotted line). The information boxes above detail the correlation coefficient (R), significance level (*p*), and regression slope (β). Shaded areas represent the 95% confidence intervals (95% CI).

**Figure 2.**
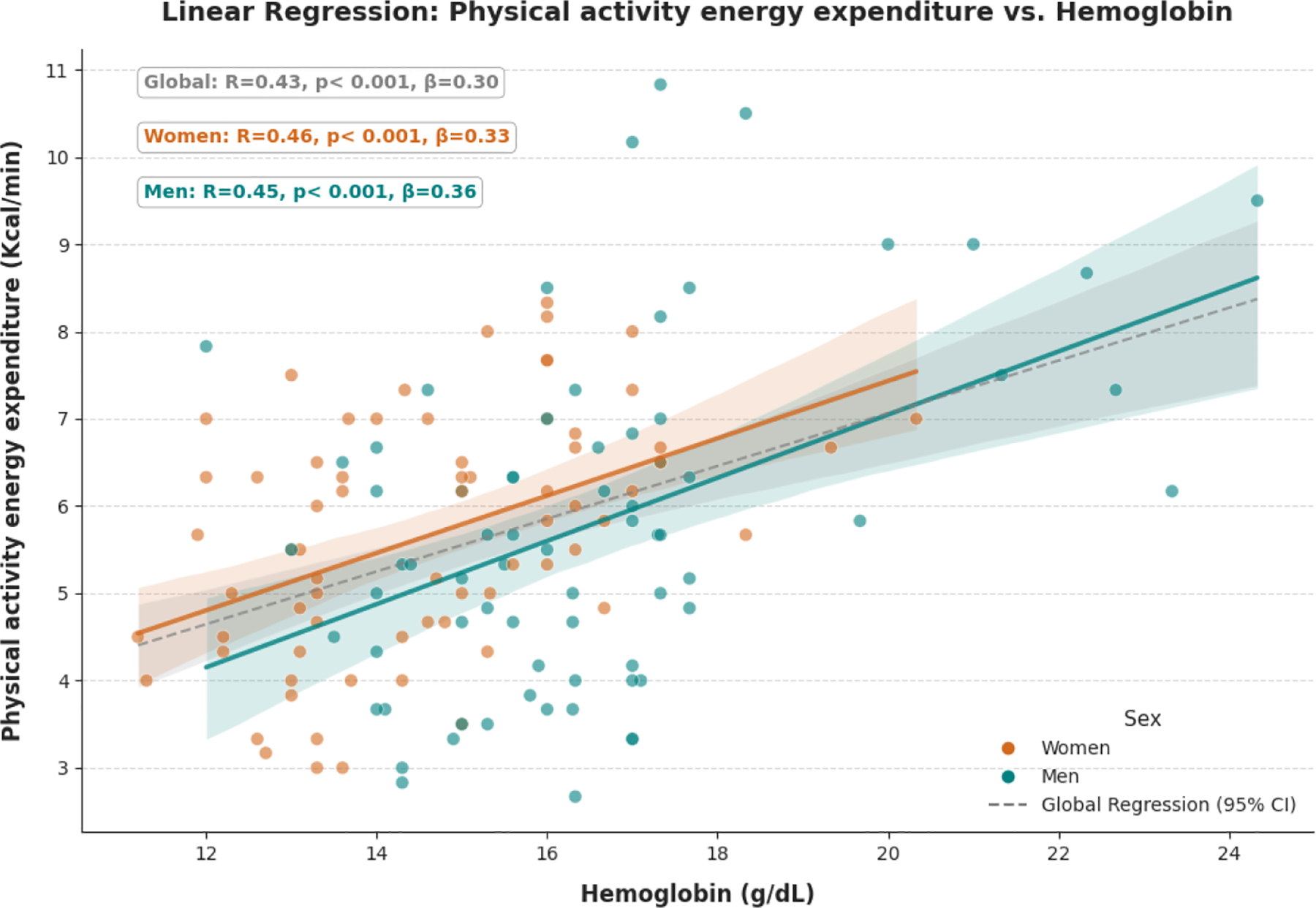
Linear Regression of PAEE vs. Hb, stratified by sex. Data are coded by sex: women (orange) and men (turquoise), along with an overall regression (gray dotted line). Shaded areas around the trend lines represent the 95% confidence intervals (95% CI).

A positive, albeit weak, relationship was identified between Hb levels and EE (R = 0.29). Overall, REE increased by approximately 0.08 kcal for every 1 g/dL increase in Hb (*p* < 0.001). This association was more pronounced in men (R = 0.39; β = 0.12) than in women (R = 0.27; β = 0.09) (Figure 1). Similarly, linear regression analysis demonstrated a significant positive correlation between Hb and PAEE (R = 0.43; *p* < 0.001; β = 0.30), with a similar response between sexes (Women: R = 0.46, β = 0.33; Men: R = 0.45, β = 0.36) (Figure 2).

**Figure 3.**
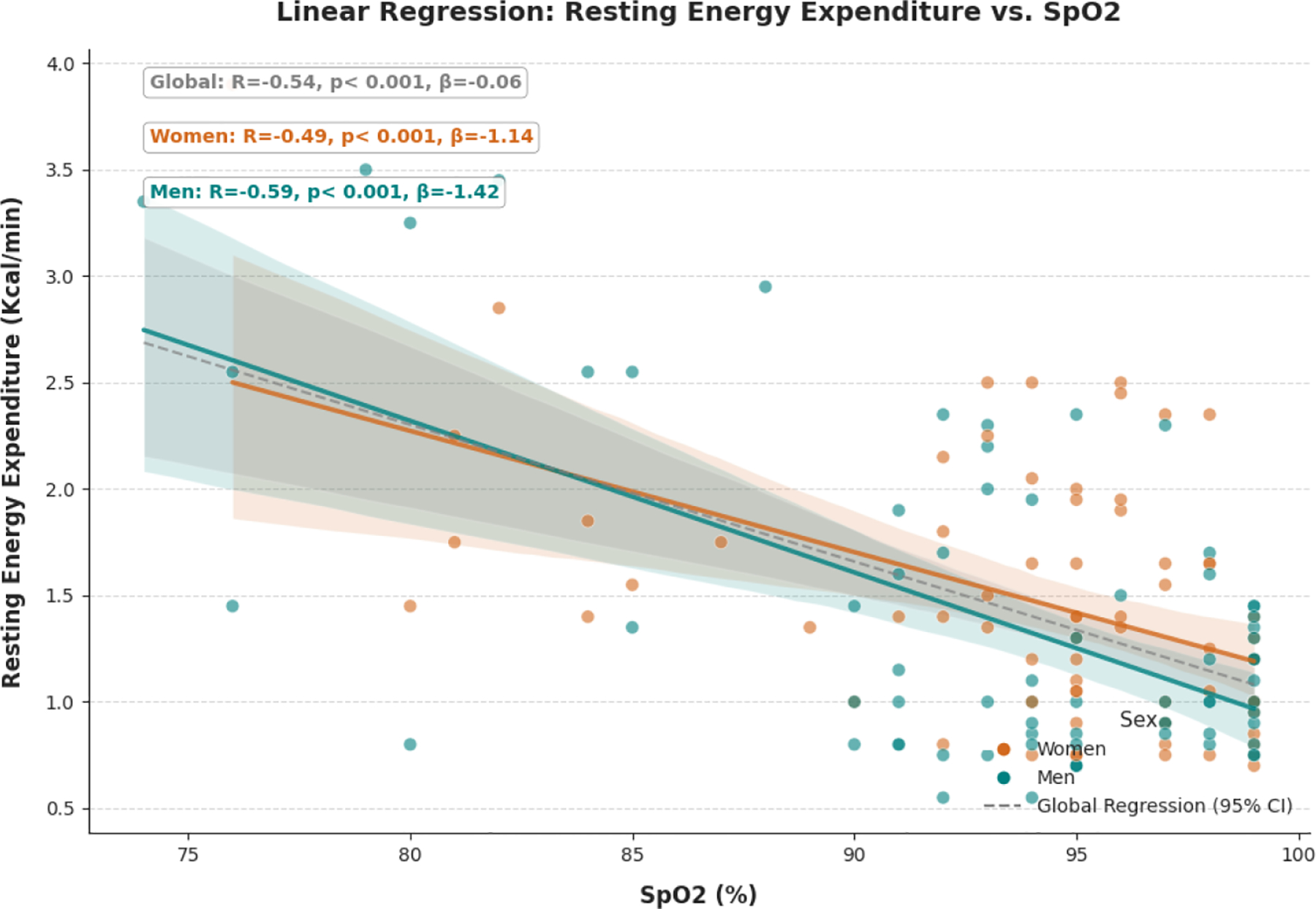
Linear Regression of Resting Energy Expenditure vs. SpO_2_, stratified by sex. Data are coded by color, women (orange) and men (turquoise), along with an overall regression (gray dotted line). Correlation parameters (R^2^, *p*-value, and β) are detailed in the information boxes above. Shaded areas around the lines represent the 95% confidence intervals (95% CI).

**Figure 4.**
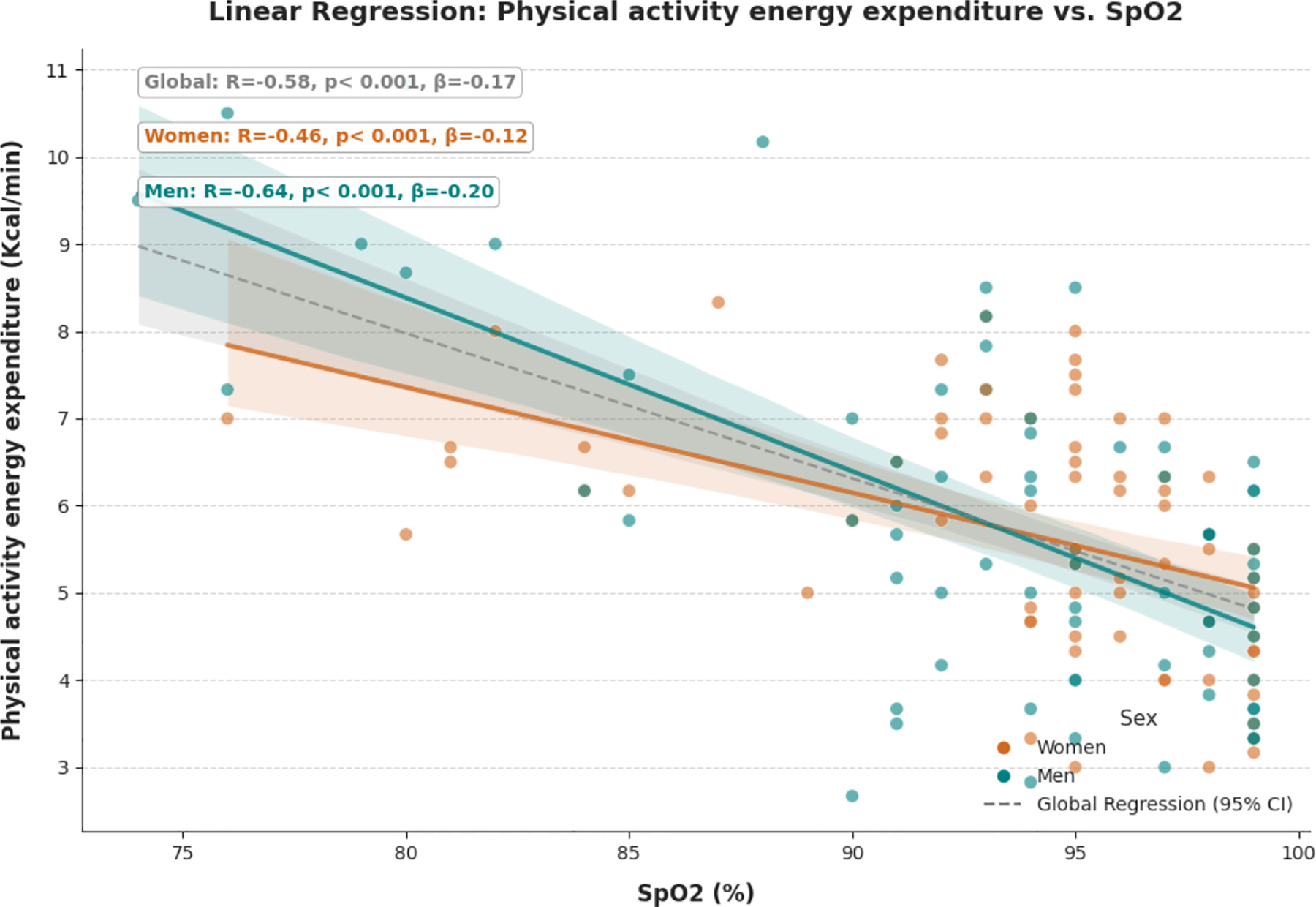
Linear Regression of Physical activity energy expenditure vs. SpO_2_, stratified by sex. Data are coded by sex: women (orange) and men (turquoise), along with an overall regression (gray dotted line). Shaded areas around the lines represent the 95% confidence intervals (95% CI).

Conversely, SpO_2_ showed a significant negative relationship with REE. For every 1% increase in SpO_2_, REE decreased by approximately 0.06 kcal/min (R = *−*0.54; *p* < 0.001), with the correlation being stronger in men (R = *−*0.59) than in women (R = *−*0.49) (Figure 3). Under physical activity conditions, SpO_2_ explained 33.1% of the variability in PAEE (R^2^ = 0.331; *p* < 0.001), with a regression coefficient of 0.17, indicating that for every unit increase in SpO_2_, caloric expenditure decreased by approximately 0.17 kcal/min (Figure 4). When stratified by sex, this inverse association was found to be stronger in the group of men (R = *−*0.64) compared to that of women (R = *−*0.46) (Figure 4).

**Figure 5.**
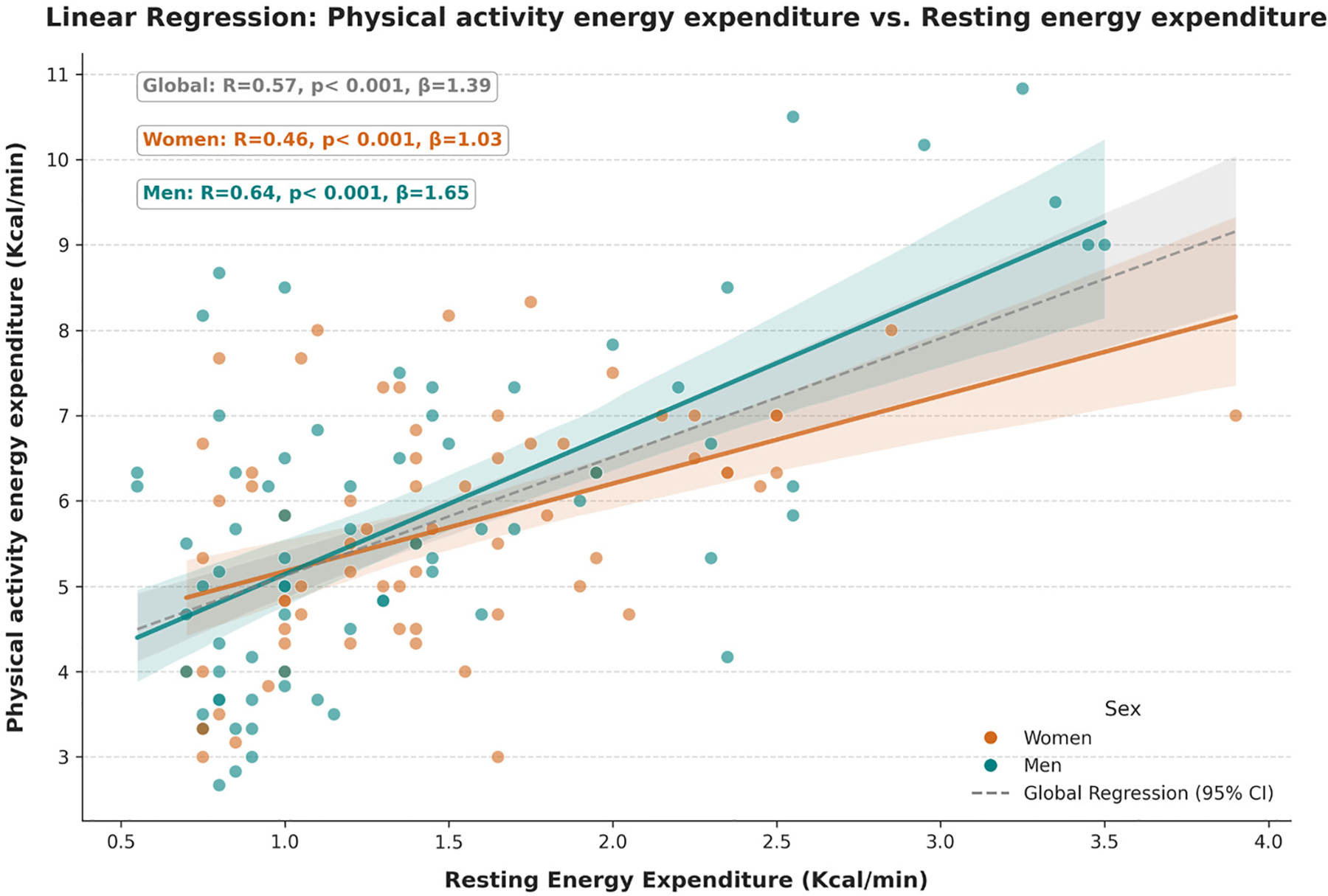
Linear Regression of REE vs. PAEE. Data are coded by sex: women (orange) and men (turquoise), along with an overall regression (gray dotted line). Shaded areas around the lines represent the 95% confidence intervals (95% CI).

Finally, the relationship between REE and PAEE was analyzed as a function of altitude. A moderate (R = 0.57) but statistically significant (*p* < 0.001) positive correlation was found, indicating that subjects with higher resting metabolic rates also tend to exhibit higher EE during exertion (Figure 5).

## 4. Discussion

This study provides the first comparative evaluation of resting and PAEE across a comprehensive altitudinal gradient, characterizing the metabolic impact of chronic hypobaric hypoxia exposure.

The principal finding demonstrates that chronic residence in extreme high-altitude environments is associated with a marked state of systemic hypermetabolism, evidenced by a progressive increase in EE both at rest and during physical exertion. However, the ANCOVA revealed that this elevated caloric cost is neither a direct nor isolated consequence of decreased barometric pressure. After adjusting metabolic variations for demographic and anthropometric covariates, the independent effect of the altitudinal gradient lost statistical significance. This indicates a physiological collinearity: the “energetic penalty” observed in populations subjected to severe hypoxia, such as La Rinconada, represents the clinical expression of a complex interplay between environmental stress and the biological compensatory effort, cardiovascular and hematological, that each individual must deploy to preserve systemic homeostasis.

Although REE typically declines gradually with age, the elevated EE observed in the La Rinconada cohort, despite it being the oldest group, highlights a notable metabolic shift. This finding suggests that the metabolic demand imposed by chronic severe hypoxia at 5100 m exerts a dominant influence, potentially outweighing the subtle effects of age-related decline. Consequently, ambient PO2 appears to be a primary metabolic driver in these extreme environments [16,29].

This hypoxic drive manifests directly in the oxygenation and hematological profiles: SpO_2_ and Hb values exhibited a pronounced variation across the hypoxic gradient, reflecting the obligate physiological response to the reduction in the partial pressure of inspired oxygen (PIO2). At extreme altitudes such as La Rinconada (5100 m), the nonlinear relationship between altitude and barometric pressure exacerbates tissue hypoxia, substantially elevating the baseline physiological load [30–32].

Despite many studies, standardized reference ranges for normal SpO_2_ in high-altitude populations remain established [30,33]. This limitation hinders physiological assessment in these environments; thus, our findings may help establish clinical and physiological reference parameters for Andean high-altitude residents.

Consistent with physiological adaptations to hypoxia, altitude drove a significant increase in Hb concentration to optimize oxygen transport [31,34]. Our data indicate that Hb rises by approximately 1 g/dL when ascending from high (3827 m) to extreme altitudes (5100 m), reflecting a critical response to preserve tissue oxygenation [34]. However, the concentrations above 19 g/dL observed in La Rinconada residents exceed the diagnostic threshold for excessive erythrocytosis [35]. Because established literature indicates that this condition exponentially increases blood viscosity and alters hemodynamic stability, such hyperviscosity plausibly raises the baseline physiological effort, representing a key underlying mechanism for the heightened metabolic demand detected in this cohort [36]. Overall, the magnitude of these hematological and oxygenation alterations reflects a physiological burden directly proportional to the hypoxic gradient, reinforcing the interdependence among SpO_2_, Hb, and EE [37].

Concurrently, the observed variations in MAP across this gradient are consistent with the chronic stimulation of peripheral chemoreceptors induced by severe hypoxemia. As described in the literature, this stimulus triggers a sustained sympathetic vasoconstrictor discharge [9,38], a mechanism that would organically link the hemodynamic response to the elevated systemic metabolic demand evidenced in our cohort.

The internal validity of these findings is informed by the assessment of habitual physical activity levels using the short version of the IPAQ [19]. As the entire sample reported light or moderate activity levels, the potential influence of chronic training as a confounding factor, which typically alters REE and PAEE [18,39], was mitigated (Table 3). This suggests that the marked metabolic dimorphism observed at 5100 m represents an intrinsic physiological response to chronic severe hypoxia rather than differences in lifestyle.

In an initial unadjusted evaluation, the progressive increase in REE along the altitudinal gradient aligns with literature associating chronic hypoxia with heightened basal metabolic activity [40,41]. Clinically, this phenomenon has been posited as a paradoxically protective factor against the prevalence of metabolic syndrome in Andean populations. However, the results of our multivariate model profoundly nuance this premise. By demonstrating that barometric pressure does not act as an isolated statistical predictor, the data indicate that the apparent thermodynamic cost of hypobaria cannot be disentangled from the combined influences of age, body composition, and population-specific traits. Therefore, the hypermetabolic phenotype at extreme altitudes emerges not as a passive environmental effect, but as the result of a complex, indivisible interplay between environmental hypoxic stress and the individual biological profile [10].

Physiologically, the increase in basal EE reflects the sustained burden of acclimatization, driven by heightened cardiorespiratory work and chronic sympathoadrenal hyperactivity [42]. At the cellular level, literature indicates this response is mediated by hypoxia-inducible factor 1-alpha (HIF-1α), which promotes metabolic reprogramming toward glycolysis [43]. Although this pathway is a crucial survival strategy under oxygen deprivation, it is energetically inefficient, creating what has classically been considered a “calorie sink” that predisposes Andean populations to a negative energy balance [44–49]. However, our data reveal a fundamental clinical discrepancy: higher altitudes were paradoxically associated with an increased body mass index (BMI). This divergence suggests that the metabolic attrition typically induced by severe hypoxia is being countervailed by the nutritional transition and contemporary socio-demographic shifts. In mining settlements such as La Rinconada, lifestyle and labor-related factors appear to exert a more decisive influence on body composition than the inherent thermodynamic cost of extreme hypoxemia, underscoring the need to re-evaluate classical high-altitude metabolic models in these communities [16,29].

A key clinical finding is the marked increase in PAEE at altitudes above 5100 m. The metabolic parity observed between Lima and Arequipa suggests the existence of an “altitude metabolic threshold” at approximately 2500 m, below which basal compensatory mechanisms effectively maintain an energy cost equivalent to that at sea level [7]. However, upon crossing this threshold, thermodynamic efficiency sharply declines and the cost of movement increases dramatically, driven by a massive adrenergic response that accelerates substrate consumption and exacerbates cardiovascular effort during exertion [9,38,50–52].

Regarding sex-based variations, the resting energy analysis revealed a pattern of metabolic parity across most of the altitudinal gradient, consistent with previous studies in Andean natives where physiological adaptation lacks overt baseline sexual dimorphism [53,54]. The isolated exception was recorded in the Arequipa cohort, where women exhibited higher EE. Clinically, this divergence is likely attributable to sociocultural or body composition variables specific to that subsample rather than an inherent biological factor of altitude [55], although hormonal fluctuations and variations in resting ventilation may modulate these specific discrepancies [56,57]. However, our findings identify a physiological tipping point at La Rinconada (5100 m), where males exhibited a substantially higher PAEE than females. This divergence indicates that extreme hypoxia disrupts the metabolic parity observed at lower altitudes, consistent with previous reports [58].

The relative energy stability documented in women at this extreme altitude suggests greater metabolic efficiency under severe physiological stress. This advantage is likely mediated by estrogens, which exert a protective effect on mitochondrial function by enhancing lipid oxidation, thereby reducing reliance on energetically inefficient glycolytic pathways compared to men [59,60]. Conversely, the elevated metabolic cost in men is largely attributable to their heightened erythropoietic response. Males exhibit significantly higher Hb levels that induce blood hyperviscosity, which in turn increases peripheral vascular resistance and myocardial workload during exercise. In men, this mechanical burden is further compounded by a more pronounced sympathoadrenal response and greater respiratory effort, ultimately driving up total EE [61–64].

Although Hb is essential for sustaining mitochondrial oxidative phosphorylation, its overall association with REE was statistically weak. This suggests that the isolated hematological profile is not the primary biological determinant of basal metabolic demand; rather, factors such as fat-free mass and adaptive tissue-level mitochondrial efficiency likely play more prominent roles [65,66]. Nevertheless, the hypoxia-induced increase in Hb exerts a quantifiable and clinically critical metabolic cost once a specific threshold is crossed. Notably, caloric expenditure increases exponentially in subjects with Hb concentrations exceeding 18 g/dL [48]. This blood hyperviscosity imposes a direct mechanical overload on the myocardium, requiring increased cardiac work and ventricular wall tension to maintain adequate peripheral perfusion [61]. When stratified by sex, the association between Hb and REE proved to be stronger in women. This heightened metabolic sensitivity to Hb variations in the female phenotype reinforces the premise that compensatory responses are intricately modulated by sex-specific factors in high-altitude environments [66,67], consolidating excessive erythrocytosis as a primary stressor that drives up energy demand under chronic hypoxia.

During physical exertion, the theoretical advantage of optimized oxygen-carrying capacity (CaO_2_) provided by elevated Hb [68,69] is immediately overshadowed by its mechanical toll. Rather than simply facilitating a more vigorous metabolic flux, hematological compensation under extreme conditions like La Rinconada exacerbates blood hyperviscosity, exponentially raising peripheral vascular resistance and the myocardial work required to maintain adequate tissue perfusion [64,70]. Consequently, this mechanical strain, coupled with a massive release of catecholamines, accelerates oxidative metabolism to compensate for the reduced thermodynamic efficiency. Although men present higher absolute levels of fat-free mass and EE, often reaching thresholds where cardiac output becomes markedly inefficient due to this exponential increase in viscosity, the underlying correlation indicates that this mechanical penalty critically impairs the thermodynamic economy of both sexes [63]. Interestingly, our stratified analysis revealed that the female phenotype demonstrates a tighter metabolic dependence on fluctuations in oxygen transport capacity during exertion. Nevertheless, the moderate magnitude of this association underscores that the hematological profile is only one of multiple determinants modulating caloric expenditure. Evidently, other mechanisms, such as adaptive tissue-level mitochondrial efficiency and individual heterogeneity in the hypoxic ventilatory response, play equally decisive roles in the comprehensive energy balance of high-Andean residents [56].

Conversely, shifting the focus from oxygen transport to availability, a clinically relevant inverse association was identified between peripheral SpO_2_ and REE. As tissue oxygen bioavailability decreases under chronic hypobaric hypoxia, it inherently compromises the efficiency of oxidative processes [35]. To sustain cellular viability amidst this deficit, the organism is compelled to rely on less efficient metabolic pathways but is also compelled to increase baseline cardiorespiratory work [52,71]. In extreme altitude environments such as La Rinconada, this substantial acclimatization cost translates into a profound systemic physiological strain, which is clinically captured by the sustained elevation in resting HR that directly drives up overall caloric expenditure [72].

Quantitatively, each 1% reduction in SpO_2_ was associated with an approximate increase of 1.286 kcal in REE. This elevated cost reflects a profound sympathetic activation, an obligate response that comprehensively accelerates basal metabolism to counteract the hypoxic deficit [9,63]. Crucially, this inverse correlation proved to be statistically stronger in women. This phenotypic distinction is likely mediated by the complex interaction of sex hormones with mitochondrial function, ventilatory control, and enhanced peripheral chemoreceptor sensitivity to oxygen gradients [36,59].

Similarly, during physical activity, an inverse and clinically profound association was identified between SpO_2_ and PAEE. This finding evidences an almost linear “compensation cost,” wherein each percentage drop in saturation demands a proportional increase in metabolic expenditure to preserve cellular viability and physical performance [73]. Physiologically, this translates into a marked loss of thermodynamic efficiency. The higher caloric requirement observed in high-altitude residents is not driven by a greater capacity for mechanical work, but rather by a massive reallocation of energetic resources toward autonomic compensatory processes. In response to exercise-induced hypoxemia, peripheral chemoreceptors trigger a critical sympathoadrenal response, with catecholamine levels in extreme environments like La Rinconada potentially doubling those at sea level [74,75] which drastically intensifies cardiorespiratory workloads. Consequently, the organism is compelled to consume a substantial energy surplus to achieve the exact same mechanical workload as at lower altitudes [76].

The consistency of this clinical relationship suggests that, even in native residents with robust hematological adaptations, SpO_2_ remains the primary indicator of metabolic effort at extreme altitudes. Although factors such as mechanical efficiency and ambient temperature contribute to overall energy balance, the arterial oxygen deficit acts as the critical stressor that preserves homeostasis at the expense of a drastic increase in caloric cost [11].

The biological plausibility of this elevated demand is definitively supported by the functional dissociation observed during the 6MWT. Despite achieving equivalent mechanical performance (distance covered) across all altitudes, post-exertional HR and PAEE were significantly higher in the hypoxic cohorts. This disparity confirms a profound degradation of thermodynamic efficiency: in the face of severe hypoxemia, the organism is compelled to divert a substantial proportion of its caloric expenditure toward sustaining cardiorespiratory viability, sacrificing the energetic economy typically required to produce external mechanical work [28,76,77]. Consequently, the elevated cost of movement at 5100 m embodies the inescapable ‘energetic penalty’ necessary to ensure systemic survival under chronic oxygen restriction [74,75].

Regarding the interaction between metabolic states, a clinically and statistically significant correlation was identified between REE and PAEE. This finding confirms that basal metabolism acts as a physiological “floor” determining the magnitude of energy demand during exertion. This linear relationship underscores the systemic nature of the metabolic burden imposed by extreme altitude, indicating that subjects with heightened basal requirements inherently exhibit a disproportionately higher caloric cost during movement.

As observed in the resting state, the ANCOVA for PAEE revealed that the independent effect of geographic location exhibited a marginal trend once adjusted for age, sex, and BMI. This suggests that extreme altitude acts as a distal environmental determinant whose metabolic influence is primarily expressed through intermediate biological mediators, such as the chronotropic response, peripheral SpO_2_, and compensatory erythropoiesis. Consequently, the loss of statistical significance for the city as a categorical factor reflects a profound physiological collinearity: as individual anthropometric and cardiorespiratory variables are adjusted, they absorb the variance inherently driven by chronic hypoxic exposure [10,37,50].

In summary, under conditions of severe hypobaric hypoxia, the organism adopts a state of sustained hypermetabolism driven by continuous chemoreceptor activation and the resulting increase in cardiorespiratory workload [75]. Therefore, physical exertion does not occur in isolation but is superimposed on an already exacerbated energetic baseline. This state of sympathetic alertness and adrenergic response imposes a systemic caloric “penalty,” wherein the elevated cost of compensatory vital functions is added to the demands of mechanical work, thereby drastically impairing the overall thermodynamic economy of any physical action at these critical altitudes [43,52,78].

### Limitations

The primary technical limitation of this study lies in the estimation of EE using wearable technology (Kalenji HR500), a method based on the linear relationship between HR and VO_2_. While indirect calorimetry remains the gold standard, its implementation at an altitude of 5100 m presents insurmountable logistical barriers [79]. Although more technically limited, this method was selected because it allowed us for direct application across all study locations. Furthermore, the protocol restricted moderate exercise to only two hours prior to assessment, whereas a period of 12 to 24 h is ideal, and lacked a prior visit to objectively assess participants’ physical activity habits. Nevertheless, given the reliability of PPG sensors for monitoring physiological effort trends in challenging environments [80], the use of the same device under controlled conditions minimized inter-device bias.

## 5. Conclusions

While an initial unadjusted analysis suggested a gross increase in EE across the altitudinal gradient, our findings demonstrate that there is no independent relationship between REE and the altitude of residence. The ANCOVA confirms that covariates, such as body mass index (BMI), negate the initial relationship. Therefore, altitude does not act as an isolated metabolic predictor; rather, the variations in EE are primarily explained by the demographic and anthropometric phenotype of the individuals. The city of La Rinconada (5100 m) delimits a critical clinical threshold where the loss of thermodynamic efficiency reaches its maximum expression. At this altitude, maintaining systemic homeostasis requires an “energetic penalty” primarily mediated by progressive peripheral desaturation. Erythropoietic overcompensation represents a limiting biomechanical burden. Hb levels exceeding 18 g/dL exponentially increase myocardial work due to blood hyperviscosity. It is physiologically relevant that this association between the hematological profile and EE is statistically stronger in women, evidencing a critical sexual dimorphism in compensation strategies against hypoxic stress. Despite the substantial increase in cardiovascular and metabolic effort, mechanical performance, assessed by the distance covered in the 6MWT, shows no significant variations compared to sea level. This clinically demonstrates that the organism adapted to severe hypoxia prioritizes systemic viability and function over energy economy.

## Figures and Tables

**Table 1.** Anthropometric variables of participants according to their altitude of residence. Comparisons among the four altitude groups (Lima, Arequipa, Puno, and La Rinconada) were performed using one-way ANOVA. Reported *p*-values represent global comparisons across cities, stratified by sex for each variable.

	Women					Men				
	Lima N = 40	Arequipa N = 20	Puno N = 21	La Rinconada N = 09	*p* Value	Lima N = 20	Arequipa N = 20	Puno N = 21	La Rinconada N = 10	*p* Value
Age (years)	21.9 ± 1.6	22.3 ± 2.5	23.6 ± 1.9	27.6 ± 5.3	0.000 *	20.9 ± 2.4	22.8 ± 2.1	24.1 ± 2.6	34.2 ± 2.6	0.000 *
BMI (kg/m^2^)	24.7 ± 2.8	24.8 ± 2.1	24.1 ± 3.1	25.9 ± 5.8	0.523	24.4 ± 2.9	25.6 ± 2.8	24.7 ± 3.9	28.0 ± 4.2	0.043 *

*, statistically significant (*p* < 0.050); BMI, body-mass-index.

**Table 2.** Physiological variables of participants according to altitude. Values are presented as estimated marginal means ± standard error, adjusted for age and body mass index (BMI) using an analysis of covariance (ANCOVA). Reported *p*-values represent the global comparison across cities for each variable after covariate adjustment.

	Women					Men				
	Lima	Arequipa	Puno	La Rinconada	*p* Value	Lima	Arequipa	Puno	La Rinconada	*p* Value
SpO_2_ (%)	98.6 ± 0.4	95.6 ± 0.4	93.0 ± 0.4	81.9 ± 0.6	<0.001 *	98.0 ± 0.4	95.0 ± 0.4	92.4 ± 0.4	81.3 ± 0.7	<0.001 *
HR (bpm)	72.2 ± 1.6	80.1 ± 1.5	77.0 ± 1.5	83.3 ± 2.7	<0.001 *	69.1 ± 1.5	77.0 ± 1.5	73.9 ± 1.4	80.2 ± 2.8	0.350 *
Hb (g/dL)	13.2 ± 0.3	13.7 ± 0.2	15.4 ± 0.2	18.3 ± 0.4	<0.001 *	15.0 ± 0.2	15.5 ± 0.2	17.2 ± 0.2	20.1 ± 0.4	<0.001 *
Hct (%)	39.4 ± 0.7	41.3 ± 0.7	46.0 ± 0.7	55.1 ± 1.2	<0.001 *	45.0 ± 0.7	46.8 ± 0.7	51.6 ± 0.7	60.7 ± 1.3	<0.001 *
SBP (mmHg)	103.3 ± 2.2	99.2 ± 2.0	102.2 ± 1.9	104.9 ± 3.6	0.096	114.7 ± 2.1	110.6 ± 2.0	113.6 ± 1.9	116.4 ± 3.8	0.970
DBP (mmHg)	71.6 ± 2.0	64.8 ± 1.9	65.9 ± 1.8	71.8 ± 3.3	0.003 *	77.7 ± 1.9	70.9 ± 1.8	72.0 ± 1.8	77.9 ± 3.4	0.688
MAP (mmHg)	82.2 ± 1.9	76.3 ± 1.7	78.0 ± 1.7	82.9 ± 3.0	0.006 *	90.0 ± 1.8	84.1 ± 1.7	85.9 ± 1.6	90.7 ± 3.2	0.786

*, statistically significant (*p* < 0.05); SpO_2_, oxygen saturation; Hb, hemoglobin; Hct, hematocrit; HR, heart rate; SBP, systolic blood pressure; DBP, diastolic blood pressure; MAP, mean arterial pressure.

**Table 3.** Median Metabolic Equivalent of Task (MET-minutes/week) by Gender and Altitude across Four Peruvian Cities (IPAQ Results). Comparisons between altitude groups within each sex were performed using the Kruskal–Wallis test. *p*-value correspond to overall comparisons between cities. IR, interquartile range.

Sex	Lima Median (IR)	Arequipa Median (IR)	Puno Median (IR)	La Rinconada Median (IR)	*p*-Value
Men	1040 (850–1250)	1060 (870–1230)	1050 (840–1260)	1030 (860–1240)	0.997
Women	940 (750–1100)	950 (760–1120)	930 (740–1130)	960 (770–1100)	

**Table 4.** Comparison of Resting Energy Expenditure (kcal/min) by Sex and Altitude across Four Cities.

City	Women (Mean ± SD)	Men (Mean ± SD)	*p* Value (*t*-Test)
Lima	1.04 ± 0.26	1.11 ± 0.27	0.393
Arequipa	1.65 ± 0.50	1.27 ± 0.58	0.029 *
Puno	1.46 ± 0.58	1.25 ± 0.66	0.278
La Rinconada	2.08 ± 0.82	2.48 ± 0.97	0.348

*, statistically significant (*p* < 0.05); *p* value, level of statistical significance. Sex comparisons within each city were conducted using independent samples Student’s *t*-tests.

**Table 5.** ANCOVA results for REE. An analysis of covariance (ANCOVA) was conducted to evaluate differences in REE across cities, adjusting for age, sex, and body mass index (BMI) as covariates.

Source of Variation	df	F	*p*-Value
Adjusted Model	15	4.966	<0.001
City	3	0.400	0.753
Age	1	0.483	0.488
Sex	1	0.059	0.808
BMI	1	0.200	0.656

**Table 6.** ANCOVA for Physical Activity Energy Expenditure. An ANCOVA was conducted to evaluate differences in PAEE across cities, adjusting for age, sex, and body mass index (BMI) as covariates.

Source of Variation	df	F	*p*-Value
Overall Model	15	8.050	<0.001
City	3	2.403	0.071
Age	1	1.509	0.222
Sex	1	0.124	0.726
BMI	1	0.097	0.756

**Table 7.** Comparison of Energy Expenditure during physical activity (kcal/day) by Sex and Altitude across Four Cities. Mean values ± SD. Comparisons of distance covered and HR were performed using one-way ANOVA. Sex-based comparisons for PAEE were conducted using independent samples Student’s *t*-tests.

City	6MWT		HR After Physical Activity		Energy Expenditure During Physical Activity		
	Distance (m)	*p*	HR (beats/min)	*p*	Women (kcal/min)	Men (kcal/min)	*p* Value
Lima	339.8 ± 20.1	0.830	92.1 ± 9.3	0.001 *	4.44 ± 0.86	4.70 ± 1.02	0.391
Arequipa	342.1 ± 14.8		97.7 ± 13.6		5.54 ± 1.35	5.13 ± 1.45	0.363
Puno	338.5 ± 18.2		102.4 ± 12.0		6.46 ± 0.97	6.17 ± 1.82	0.520
La Rinconada	340.2 ± 15.5		107.6 ± 9.8		6.80 ± 0.87	8.43 ± 1.69	0.018 *

*, statistically significant (*p* < 0.05); 6MWT, 6-min walk test; HR, heart rate.

## Data Availability

The data are available; please contact the authors if you require them, and you will be able to access this information.
